# Group Cohesion and Necessary Adaptations in Online Hearing Voices Peer Support Groups: Qualitative Study With Group Facilitators

**DOI:** 10.2196/51694

**Published:** 2024-05-03

**Authors:** Alison Branitsky, Eleanor Longden, Sandra Bucci, Anthony P Morrison, Filippo Varese

**Affiliations:** 1 Division of Psychology and Mental Health, School of Health Sciences Faculty of Biology, Medicine and Health The University of Manchester Manchester United Kingdom; 2 Psychosis Research Unit Greater Manchester Mental Health NHS Foundation Trust Manchester United Kingdom; 3 Complex Trauma and Resilience Research Unit Greater Manchester Mental Health NHS Foundation Trust Manchester United Kingdom; 4 Manchester Academic Health Science Centre The University of Manchester Manchester United Kingdom

**Keywords:** peer support, group cohesion, web-based delivery, hearing voices, Hearing Voices Movement, self-help groups

## Abstract

**Background:**

Face-to-face hearing voices peer support groups (HVGs), a survivor-led initiative that enables individuals who hear voices to engage with the support of peers, have a long-standing history in community settings. HVGs are premised on the notion that forming authentic, mutual relationships enables the exploration of one’s voice hearing experiences and, in turn, reduces subjective distress. As such, group cohesion is assumed to be a central mechanism of change in HVGs. The rise of digital mental health support, coupled with the COVID-19 pandemic, has resulted in many HVGs adapting to online delivery. However, to date no studies have examined the implementation of these online groups and the adaptations necessary to foster cohesion.

**Objective:**

This study aims to understand the experience of group cohesion among HVG facilitators in online groups compared with face-to-face groups. Specifically, we examined the ways in which the medium through which groups run (online or face-to-face) impacts group cohesion and how facilitators adapted HVGs to foster group cohesion online.

**Methods:**

Semistructured qualitative interviews were conducted with 11 facilitators with varied experience of facilitating online and face-to-face HVGs. Data were analyzed using reflexive thematic analysis.

**Results:**

The findings are organized into 3 themes and associated subthemes: nonverbal challenges to cohesion (*lack of differentiation*, *transitional space*, *inability to see the whole picture*, and *expressions of empathy*); discursive challenges to cohesion (*topic-based conversation* and *depth of disclosure*); and necessary adaptations for online groups (*fostering shared experience* and *using the unique context to demonstrate investment in others*). Despite challenges in both the setting and content of online groups, facilitators felt that group cohesion was still possible to achieve online but that it had to be facilitated intentionally.

**Conclusions:**

This study is the first to specifically investigate group cohesion in online HVGs. Participants noted numerous challenges to group cohesion when adapting groups to run online, including the unnaturally linear narrative flow of dialogue in online settings; lack of transitional spaces, and associated small talk before and after the session; ease of disengagement online; inhibited sharing; and absence of shared physical presence online. Although these challenges were significant, facilitators nevertheless emphasized that the benefits provided by the accessibility of online groups outweighed these challenges. Necessary adaptations for cultivating group cohesion online are outlined and include capitalizing on moments of humor and spontaneity, using group activities, encouraging information sharing between participants using the chat and screen-sharing features, and using objects from participants’ environments to gain deeper insight into their subjective worlds.

## Introduction

### Background

Hearing voices peer support groups (HVGs) have a long-standing history within the community [1]. The establishment of HVGs is one of the primary objectives of the Hearing Voices Movement (HVM), an international, survivor-led coalition of voice hearers and their allies, which strives to shift professional and public attitudes toward voice hearing away from biogenetic, pathology-based models toward understandings that locate voice hearing as a complex psychological experience imbued with subjective meaning (eg, psychosocial, cultural, or spiritual) [1]. HVGs are premised on the notion that forming genuine, mutual relationships between group members can support individuals to safely explore their voice hearing experiences and, as such, may result in positive psychosocial change [2-11]. As a result, group cohesion is posited to be of central importance in HVGs [7,9].

The concept of group cohesion arises from group psychotherapy literature and refers to participants’ feelings of connectedness to one another and to the group as a whole [12]. Group cohesion is believed to be the foundational feature upon which all other therapeutic work takes place [12]. Although their distinct ideologies mean theories of group psychotherapy cannot be uncritically applied to peer support groups [13], it may nevertheless be instructive to further investigate the role of group cohesion in HVGs.

Although peer support is an established aspect of mental health provision, a more recent development concerns their online delivery; a process largely expedited by the COVID-19 pandemic, wherein many forms of support, including HVGs, were forced to be held remotely. As a result, there has been growing interest in understanding if and how group cohesion can form in online groups [14-16]. The literature on online group cohesion has been largely theoretical and anecdotal but suggests that although group cohesion does occur, there are various challenges to its achievement, including the ease of disengagement and the lack of embodied presence of both the participants and therapist [14]. Given that online peer support represents a potentially accessible and scalable form of support that can be implemented in multiple contexts globally, it is necessary to further investigate the development of group cohesion in online spaces. This type of investigation is particularly important to HVGs as they are an existing resource that exist internationally and can provide vital support to individuals who may otherwise be insufficiently supported by mental health services [17,18].

### Study Objectives

This study aims to understand HVG facilitators’ experience of group cohesion in online groups compared to face-to-face groups. Specifically, the following issues were examined: (1) How does the medium through which groups run (online vs face-to-face) impact group cohesion? and (2) How do facilitators adapt HVGs to foster group cohesion online?

## Methods

### Design

Semistructured qualitative interviews were conducted with 11 HVG facilitators. Data were analyzed using a hybrid of deductive and inductive reflexive thematic analysis (RTA), a flexible approach that recognizes the centrality of the researcher’s subjectivity as an analytical tool and emphasizes reflective engagement with theory, data, and interpretation [19]. The study was further underpinned by a critical realist epistemological position, which acknowledges the existence of an objective social world while recognizing that one’s understanding of that social world is shaped by one’s experiences within it [20]. The subjective realities of both participants and researchers were acknowledged while still aiming to understand and explain experiences that exist beyond the study sample [21].

Topic guides were developed by AB in consultation with patient and public involvement and engagement (PPIE) experts with relevant experience of HVG facilitation or membership. Specifically, topic guides were informed by the supposition by Yalom [12] that group cohesion is the foundation for effective groups; as such, the interviews focused on facilitators’ experiences of if and how group cohesion was cultivated in both face-to-face and online settings. Topic guides were further informed by previous literature on the proposed mechanisms of action in HVGs [6] as well as the lived experience of AB and PPIE representatives as HVG facilitators. All questions were piloted with PPIE experts before commencing recruitment, with topic guides revised based on PPIE feedback. Questions were open ended and sequencing was determined based on the flow of conversation. Probes were used throughout to elaborate on relevant topics. Topic guides were iterative, with specific questions being added or emphasized as preliminary themes began to be constructed. Reflective logs were kept throughout data collection and analysis. The COREQ (Consolidated Criteria for Reporting Qualitative Research) were consulted in reporting this research (Multimedia Appendix 1) [22].

### Participants

**Figure 1:**
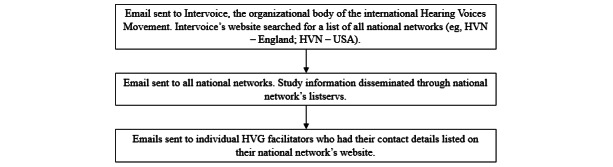
Flowchart of snowball sampling approach used to recruit hearing voices peer support group (HVG) facilitators. HVN: hearing voices network.

Participants in this study comprised an international sample of HVG facilitators, who were recruited using convenience and snowball sampling methods through the HVM (Figure 1). Adults who spoke English and had at least 3 months of experience facilitating HVGs were eligible for interview. The sample size was determined through information power [23] and thematic sufficiency [19], with the former referring to the degree in which participants hold information to address the research question and the latter referring to the point at which data collection is sufficiently complete as to meaningfully answer the research questions. This approach to data completeness was chosen over the more common idea of *data saturation*, which is subjective in nature and theoretically incompatible with RTA [24].

### Ethical Considerations

Ethics approval was received from the University of Manchester Research Ethics Committee (2022-13944-22907), and all participants provided written informed consent. Participants received a participant information sheet outlining the parameters of study involvement and were given a minimum of 24 hours to decide whether they wanted to take part. The voluntary nature of the research was emphasized throughout, including during the qualitative interviews. All interviews were confidential, and transcripts were pseudonymized with any other identifiable information removed. Due to resource constraints, no compensation was offered for study participation. Furthermore, due to the potentially identifiable nature of the data, interview transcripts were not made available via public data repositories, and the data were not shared outside the research team.

### Procedure

Potential participants contacted AB via email to express their interest in the study. Interviews were conducted by AB, a researcher with lived experience of voice hearing and HVG group facilitation, between April and June 2022. Participants were aware of AB’s background as an HVG facilitator. All interviews were conducted online via Zoom (Zoom Video Communications, Inc) [25] and were audio recorded using Zoom’s built-in encrypted recording software. Interviews were then transcribed verbatim. Participants were assigned a pseudonym, and identifying information was removed from the transcripts. The median interview length was 58 minutes 42 seconds (range 38 min 7 s to 89 min 20 s).

### Analysis

A predominantly inductive approach to data analysis was adopted. Data were open coded, and preliminary codes were inductively generated based on participants’ responses. However, given that the topic guide was framed on the theoretical assumption that group cohesion was crucial for both face-to-face and online HVGs, deductive analysis was also used to allow for the identification of codes that could meaningfully answer the research question. As such, both latent and semantic codes were generated. The iterative approach to data analysis by Braun and Clarke [19] was followed: (1) data familiarization through repeated reading of transcripts, (2) generating initial codes, (3) generating initial themes, (4) reviewing initial themes, (5) defining and naming themes, and (6) producing the report. The final thematic structure was derived by AB in consultation with EL, SB, APM, and FV. Data analysis took place using NVivo (version 12; QSR International) [26]. No attempt was made to establish interrater reliability as it is antithetical to the philosophical position of RTA [27]; however, sense checking among the research team, the use of reflective notes and memos, and the 20-point guide by Braun and Clarke [27] for the assessment of RTA research quality were used to enhance the trustworthiness of the data. Furthermore, validation of the thematic structure was sought from all study participants, all of whom consented to member checking, as well as from PPIE representatives.

### Reflexivity

All the researchers in this study hold perspectives on voice hearing that align with those of the HVM; specifically, that voice hearing is an inherently meaningful psychological experience that is worthy of ongoing exploration. AB is a PhD-level researcher; EL is an academic psychologist; and SB, APM, and FV are academic clinical psychologists; all of whom have experience supporting and developing interventions, including digital interventions, for individuals who hear voices. All researchers had extensive experience conducting and analyzing qualitative data. AB and EL have similarly been members and facilitators of HVGs (AB has experience with online and face-to-face HVGs, and EL has experience with face-to-face HVGs). The background of the research team, particularly their commitment to developing various forms of psychosocial support for voice hearers, influenced the generation and interpretation of the data. To maintain transparency around how the researchers’ backgrounds were interacting with the data, AB kept reflective logs to record impressions about interviews and emergent themes and document ways in which findings paralleled or differed from her lived experience as a facilitator. Emphasis was placed on ensuring that divergent views were represented within both analysis and reporting.

## Results

### Overview

A total of 11 participants consented to take part in the study. Facilitators ranged in age from 25 to 52 (mean 41.27, SD 7.40) years with most participants identifying as male (7/11, 64%) and White (10/11, 91%). Participants had between 1 and 10 (mean 5.45, SD 3.06) years of experience facilitating HVGs. Key participant characteristics are presented in Table 1, with a table of full participant characteristics available in Multimedia Appendix 2.

**Figure 2:**
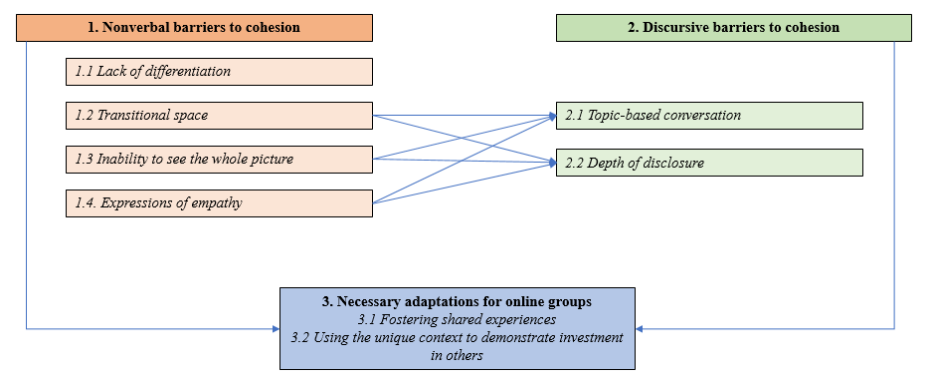
Thematic map depicting the relationships between themes and subthemes.

Results were organized into three themes and associated subthemes: (1) nonverbal barriers to cohesion (*lack of differentiation, transitional space, inability to see the whole picture,* and *expressions of empathy*), (2) discursive barriers to cohesion (*topic-based conversation* and *depth of disclosure*), and (3) necessary adaptations for online groups (*fostering shared experiences* and *using the unique context to demonstrate investment in others*). The findings from the first 2 themes informed the necessary adaptations presented in the third theme. A thematic map of the findings is presented in Figure 2.

**Table 1:** Participant characteristics.

Name	Age (y)	Gender	Geographical location	Group medium	Group moved online during the COVID-19 pandemic?	Length of time facilitating (y)
Sean	41	Man	Western Europe	Face-to-face	No	9
Annika	34	Woman	Northern Europe	Both	Yes	4
Patrick	46	Man	Western Europe	Online	No	1
Rachel	25	Woman	North America	Face-to-face	Yes	1
Arjun	36	Man	Western Europe	Both	Yes	5
Callum	52	Woman	Western Europe	Both	Yes	2
Sabina	36	Woman or gender fluid	Western Europe	Both	Yes	8
Michail	46	Man	North America	Both	Yes	5
Lex	46	Man	Western Europe	Both	Yes	10
Noah	47	Man	Western Europe	Face-to-face	No	8
Isabella	45	Woman	Western Europe	Both	Yes	7

### Nonverbal Barriers to Cohesion

Groups that took place online lacked many of the nonverbal elements of communication that were previously relied upon to create a cohesive space. These nonverbal elements included both the group setting and cues between HVG participants.

#### Lack of Differentiation

HVGs were often described as “unique” (Lex) and “different” (Rachel) spaces, whose ethos of curiosity, openness, and nonjudgmental approaches toward voice hearing were intentionally cultivated and led to connections that could not be fostered elsewhere:

It wasn’t ever said...but we all picked up that describing these sorts of [voice hearing] experiences wasn’t welcomed in other groups. So we participated in other groups on another level. But have these [voice hearing] experiences close to our chest. So when you have this space to talk about it and then realize “oh I’m so far from alone in this” that you have all these different individuals... and all of a sudden it’s kind of like, “oh you share this with me as well.” And it feels very opposite of being alone. Like, it was really connected.

Annika

In turn, many facilitators took great care to create a soothing environment that reflected the distinctness of the groups:

We had always put a lot of emphasis on making a nice atmosphere. Much more than in our [other] groups. We turned off the industrial lights; we had candles; we sat in a circle. You don’t even need to sit on a chair if you don’t want to. We had like yoga meditation chairs. And some people sat on the floor. And it was so different.... We were really thinking of the atmosphere. And then when we moved online it’s just, uh, the screen and the face and you couldn’t really make this atmosphere in the same way.

Annika

In contrast, online meetings tended to feel indistinct from both other mental health groups, as well as other online gatherings, wherein participants were often sitting in the same place regardless of whether they were joining the HVG, a family event, or a work meeting. In this regard, this lack of differentiation resulted in grief as it felt some HVGs lost the distinctiveness they had in the face-to-face setting. Groups such as Annika’s, which adhered very strongly to the ethos of the HVM, particularly struggled with the transition online because the truly distinct nature of the space was largely lost*.*

#### Transitional Space

Facilitators spoke to the importance of transitional space, or the “before and after” (Lex) of the group, where participants could “walk together to the bus stop” (Lex) or meet in the doorway over tea and coffee to enjoy small talk and forge “potential relationships outside of this room” (Annika). The social energy likewise made its way into the meeting, as described by Sabina: “Often there’s coffee or cookies or something like that and so often a conversation...it starts with...‘the coffee is stronger this time than last time’.” As such, entering a group without a transitional space could potentially feel lonelier and more jarring, especially for new members:

Whereas with online groups, particularly if you’ve never been before, it really is, it’s that all or nothing: “I’m in the waiting room by myself and now I’m with all of these people that I don’t know. And there’s not that, there’s no clear etiquette about how I’m supposed to behave because every group is completely different.”

Annika

Similarly, facilitators also reported how a lack of intentional time for small talk could inhibit members’ subsequent willingness to share, given that they no longer had initial opportunities to connect on less vulnerable topics.

#### Inability to See the Whole Picture

As described by facilitators, online groups posed a unique visual context: that rather than sitting in a circle, as is customary with face-to-face groups, participants were instead confined to small, 2D screens online, if their cameras were even on at all. As such, the information and input participants received from and about one another was diminished, which could make individuals feel less connected. As Lex described, “some people get their camera off. Only audio...that makes a whole different dynamic,” whereas Michail noted how “on Zoom it’s just so much easier to hide.” Even in cases where participants had their cameras on, engagement could still be difficult to assess, as it was impossible to tell, for example, if “you’re busy playing with your phone” offscreen (Arjun).

In this regard, one clear impact of meeting online was the ease with which participants could disengage with the group. As Michail reported, “it’s just so much easier to be distracted on Zoom.” Although seeing members in their own home enabled them to join the group from an environment that felt safe and comfortable, at times this could also be taken to the extreme when participants visibly engaged in other activities during the meeting:

[Group member] sat there and poured himself a glass of beer. And drank it during the group. And it’s like, that would have never happened if we were sitting together in a circle because it’s just, it doesn’t work like that.

Isabella

Not all facilitators felt it was problematic if group members engaged in other activities during the group, especially if doing so made it easier for them to participate. However, it did highlight the importance of discussing expectations about engagement with their respective members in a way that was unnecessary in face-to-face groups. Annika described having to create group agreements online that had not previously existed: “we had to have rules like, ‘you have to be dressed, you’re going to a meeting’ and ‘please do not be doing something else’.”

The ability to disengage could also pose unique challenges within the online context. Disagreement between members could be particularly troublesome in this regard, as abruptly leaving could prevent disagreements from being resolved within the group space: “One time there was sort of a heated discussion, one of the guys just hung up...but that was hard and kind of a bummer. So it’s easier to check out in that way” (Michail).

Facilitators further noted that at times it could be difficult to navigate confidentiality online, not least because many participants did not have access to truly private spaces. As Annika described, “Like they had the computer in the kitchen and a parent could just walk by whenever. It didn’t really feel as confidential.” This, in turn, could impact the trust that group members were able to develop with one another. Annika noted that this was particularly pronounced for group members who had not been a part of her group before it transitioned to being held online, stating, “I don’t remember somebody starting to trust online rather than in person.”

#### Expressions of Empathy

One of the greatest challenges posed by online groups was the lack of proximity between group participants. This resulted in participants’ needs not always being responded to in a way that felt appropriately compassionate:

I had a couple instances where I’d get on and somebody looks like they’re in trouble...they’re way over there. I can’t help them way over there. But if they’re in front of me, you can try and do something you know...If they’re definitely in front of you, you can try to give them a hug if it comes to it.

Patrick

Other facilitators spoke of how being in the same space and seeing and hearing the same things in one’s surroundings naturally fostered a sense of connection. As described by Lex, “there’s also the nonverbal part and being in the same place and seeing the same sun or rain outside when you’re having a break.... I think that’s really powerful.”

The importance of physical contact, such as “you shake each other’s hand or a sometimes a hug” (Lex) was important not just for expressing social bonds, but for conveying empathy and understanding toward participants who were distressed. Isabella compared the experiences in her online and face-to-face group in the following way:

Whereas online we had as well that people were like distressed or crying and there isn’t that same level of connection or compassion if that makes sense. Somehow, because there is this distance. There is this distance of not being in the, in the same room together.

Isabella

Facilitators described it as “natural” (Patrick) to interact in person, and that one could achieve a richer, more embodied experience wherein nonverbal cues provided important communicative information: “You know, just being in person, being in the presence of other people, the body language and just you know, you forget how much you get out of sitting with someone” (Michail).

### Discursive Barriers to Engagement

Without the nonverbal cues present in face-to-face meetings, facilitators noted that there were changes in both the style and content of verbal communication in online groups.

#### Topic-Based Conversation

Facilitators noted how the group medium could have a distinct impact on the flow of conversation. For example, a defining feature of face-to-face groups was topic-based conversations, or conversations that were centered on a particular issue to which everyone contributed, regardless of whether it was particularly relevant to them at the time. These conversations were marked by a “free flow” (Annika) of “collaborative interrupting” (Noah), where “you go to something and it leads to something else” (Arjun). “There’s no uncomfortable silences [and] everybody gets to speak” (Sean) and “[participants] really bounced off each other and related to each other on experiences that you wouldn’t imagine that anybody could relate on” (Rachel):

If it was running well, we really didn’t need to facilitate much...And maybe someone came in wanting to start with a, with a topic or, that they kind of add[ed] to another person’s story, but really like, it flowed.

Annika

These kinds of interactions reduced the reliance on the facilitator to keep the discussion going, with participants instead empowered to carry on the conversation themselves:

It seemed like it was definitely a running machine when everybody did kind of play a role. It definitely lessened any outlook on me as some kind of authority figure for everyone to see...And I think it made everybody feel a bit more of a sense of connection. Like it was a little community and that they all were really relating to each other.

Rachel

This “natural” (Patrick) conversation allowed discussions to deepen in a way that felt appropriate and engaging: “they respond to each other. Sometimes asking questions, deepening questions to each other” (Noah).

In contrast to face-to-face meetings, the physical distance between participants in online groups made topic-based conversation more difficult. In the absence of nonverbal cues typically present in face-to-face meeting, Isabella noted that it was difficult for participants to know when to speak next:

They have to lift the hand and say, “can I say something”...because sometimes they don’t know like, “is it my turn to speak?” “Can I speak?” Because you don’t have that connection.

This was true even in groups that transitioned to being online and where the participants had previously connected face-to-face. The resultant flow was markedly more sequential, with individuals tending to bring up a topic that was relevant to them, and the topic then shifting when the next person spoke: “Yeah, in the online setting there was, ok this topic was talked about. Now I bring this topic for new conversation” (Sabina). Thus, when stories were shared as isolated silos, participants were not connecting with each other as intimately as they would when face-to-face:

It’s more like one says something and then the other one then tells her story, the other one then tells his story and then the next one. There’s not really like interaction as much as when we were in a room together.

Isabella

#### Depth of Disclosure

Some facilitators felt that conversation could be “flattened” (Michail) online, that there was more hesitation sharing “intimate” (Sabina) details of one’s life, and that, in general, “you don’t go too heavy in the group” (Patrick). As a result, conversations sometimes lacked the depth that was often felt in face-to-face meetings. This, in turn, could hinder the development of trusting relationships because participants felt neither open enough to talk about vulnerable subjects nor secure enough to connect over day-to-day experiences. Sabina described one such situation:

In [in-]person groups, the conversation is deeper but also could often go to more lighter topics, like their favorite TV series or something like that. And in online setting it wasn’t as deep, but it wasn’t also as light. And I had the feeling like [the] participant[s], they didn’t feel secure enough to put this light things. So, they feel “ok this topic about TV series is too dumb or too, um, superficial or too, yeah whatever.” And in [in-]person group[s] they, after deep topic, they went after the connecting together, they feel secure enough to put also this more superficial [topic]. And it brings, brings a little bit of lightness and cosiness in the group setting.

### Necessary Adaptations for Online Groups

Despite the challenges posed by the online medium, many facilitators were still able to run cohesive and successful groups. This theme outlines the specific ways in which cohesion between members was cultivated online.

#### Fostering Shared Experience

Creating and building on shared experiences was a central feature of forming cohesion within online groups and was achieved in 2 primary ways: the first being capitalizing on spontaneity, and the second through group activities. In terms of the former, moments of spontaneity served as the online corollary to infectious laughter in face-to-face spaces; indeed, the fact that participants were not in a shared space could, at times, enhance the experience as they had access to objects that would not have been available within in-person groups:

A couple of times this spontaneous thing happened where one group member was wearing a new hat and he was telling us about his hat. And everybody in the group went off and got a hat. And it was so funny and fun and he still will talk about that day. And we laughed so much that day...And I remember feeling like, “it feels so good to laugh like this.” And we’ll laugh about that day still. And he wears the hat all the time and sometimes he’ll say, “c’mon guys, where are your hats? You know it’s our group. Where are your hats guys? C’mon?” You know this sort of spontaneous thing that couldn’t really happen in person.

Michail

In this respect, spontaneous, shared moments provided a sense of continuity between groups and served as a crucial moment that could be repeatedly referred to, ultimately enhancing the sense of group identity. Following this experience, Michail wondered if similar occurrences should be fostered more deliberately: “Boy I wonder if it’s nice to have an ice breaker activity where you actually share something from your space.” Similarly, such moments also enabled group members to connect with one another over other topics of mutual interest, experience, or “personal things like music” (Lex), which left facilitators feeling like they were having “a co-human kind of experience” (Lex).

Although valuable, facilitators of online HVGs also noted that moments of spontaneous humor could be few and far between. However, when this was the case, the second means of fostering shared experience could be used, namely group activities. These could range from voice-related work, such as introducing members to the Maastricht interview [28], a technique used to explore voices’ characteristics, origins, and emotional impacts; practices such as reiki; or “playing music” (Patrick) and “watching videos together” (Lex). These activities could provide a starting point for conversation: “we’ll play {Beyond Possible: How the Hearing Voices Movement Transforms Lives [29]} and it really brings up a lot of stuff to talk about” (Michail), whereas at other times, participating in the activity was a shared experience in and of itself:

Sometimes...we’ll have something like...something more structured [co-facilitator will] bring somebody in to talk about reiki or have a guest speaker for meditation or something. Um, or tapping [as described by the emotional freedom technique] and stuff like that.

Callum

The ease with which information could be shared online was an asset that offered individuals a novel way of participating in the group and sharing their experiences or interests. The chat and screen-sharing feature allowed ideas to be shared in real time, which enabled the entire group to have access to the same information. In turn, such information could be informative (“maybe if someone was sharing a visual aid or like ‘I was listening to something. Here is the link.’ It would go in the chat” [Annika]) or humorous (“if there was a funny video or something that was much easier to share in the online group” [Sabina]).

#### Using the Unique Context to Demonstrate Investment in Others

Facilitators emphasized the fact that group members were equally able to foster intimacy online as they did in face-to-face settings, but that the manner of achieving this was necessarily different. For example, seeing people in their own homes provided “an extra possibility to show an interest in their world” (Lex). At times, facilitators could gain a more contextualized sense of who a person was and what their life was like outside the group when they were able to see them in their home environments:

We had someone who wasn’t comfortable speaking, but he would attend, and he would write in the chat. And we would ask “is it okay if we read from the chat to the group” um, and, and he was okay with that. But he was doing that because he was concerned about who around him could hear.

Michail

Online groups also offered an additional level of accessibility, thus enabling individuals to take part who either did not feel comfortable participating in or did not have access to face-to-face groups. For some members, joining a group online was “a lower bar to participation” wherein “it makes it a little easier for folks who are reticent to just kind of try it” (Michail). The online medium also provided voice hearers with the opportunity to engage with the group in a way that worked best for them. For some members, this opened up new channels of communication: “if you’re not confident to speak...you can type. You’ll be able to type [in the chat]” (Callum). For others, it enabled them to engage in a way that felt safe to them:

We’ve had [participants] say “can I join and not show my face? Is that ok?” And we say...“sure, as long as it’s ok with the group, as long as the group doesn’t feel weird about it but we want you to participate as fully as you can. Or in a way that feels right for you.”...It gives a different set of opportunities for modulating engagement.

Michail

The distance and anonymity of online groups was additionally advantageous for some members, making it easier to disclose personal experiences precisely because they could talk about them in a “safe space” (Lex) that felt very distant and removed from the rest of their lives: “I would say you can very [easily] communicate stuff on Zoom you probably wouldn’t do it the person was living down the road...[online] it’s like, it’s not like it’s gonna walk out of the room” (Patrick).

## Discussion

### Principal Findings

This study is the first to explore how the medium through which HVGs are delivered impacts cohesion within the group. It is similarly the first to analyze the specific adaptations necessary to cultivate group cohesion in online peer support settings. In terms of building group cohesion, facilitators identified several nonverbal and discursive barriers to running groups online. The primary nonverbal barriers included the lack of differentiation between HVGs and other online spaces; the lack of transitional space online; the reduced visual input online, which prevented individuals from fully seeing what was going on in one another’s environment; and the inability to express and receive empathy nonverbally to the same extent as in face-to-face settings. These barriers, in turn, impacted discursive communication within the group, with online groups having an overprescriptive flow of dialogue where only one person could share at a time, thus resulting in discussions that tended to build less naturally on a single topic and where participants were discouraged from sharing as deeply. However, despite these challenges, facilitators were able to adapt to running cohesive groups online by (1) capitalizing on moments of spontaneity and using group activities to build a shared experience and (2) bringing members’ subjective environments into the group space.

These findings largely correspond with previous literature outlining how nonverbal elements of the group, both in terms of group setting and nonverbal communication between members, strongly influence group cohesion. For example, Weinberg [15] described how one of the tasks of group therapists was creating a “holding space” in which therapeutic work can take place, including deliberately choosing and arranging furniture, lighting, and objects in the room in such a way as to further therapeutic aims. Indeed, Payne et al [9] described one of the main benefits of HVGs as creating a “containing” environment in which anomalous experiences and intense emotions can be shared and processed. Similarly, facilitators in this study often took great care to create a soothing and inviting atmosphere that reflected the distinct values of HVGs; however, in the absence of this control, they could subsequently struggle to create an environment that felt sufficiently safe and containing to encourage disclosure and cohesion between members.

In turn, Weinberg [15] further noted how the opportunity for small talk before, during, and after meetings was necessary for establishing group cohesion. HVG facilitators similarly described how a lack of transitional space into and out of the group inhibited this small talk and acted as a barrier to connection. This is perhaps surprising given previous research on HVGs, which suggests that members primarily connect over the shared experience of voice hearing [4,7,30]. Future online HVGs, and online peer support groups more broadly, may therefore benefit from prioritizing time for these more informal, “co-human” (Lex) connections. This may be achieved by intentionally building in transitional space: for example, by opening the online room early and allowing participants to stay for several minutes after the group ends, encouraging small talk between participants, and having refreshment breaks during the group.

The flattening of visual cues online has similarly been demonstrated to be a barrier to cohesion. Within individual therapeutic settings, Grondin et al [31] argued that empathy and, by extension, alliance (the individual corollary to cohesion), are established through an iterative cycle of producing and perceiving cues that enable the interpretation of others’ emotional states. Although these cues can be verbal [32], they are very often nonverbal and include signals such as facial expression [33], body posture [34], and eye contact [35]. In online settings, these cues tend to be diminished [15], especially in instances where participants have their cameras off and thus, visual cues are neither sent nor received. This lack of visual cues can result in individuals feeling as though they are not being sufficiently empathically received by others [31,35], as well as having the potential to undermine trust and disclosure through reduced awareness as to whether other members are in a truly confidential space [15]. This study is largely consistent with these findings, with facilitators additionally noting the importance of overt nonverbal interactions, such as handshakes or hugs, in the establishment of cohesion. Interestingly, these barriers were still present in HVGs that transitioned from face-to-face during the COVID-19 pandemic, with facilitators often describing highly cohesive groups that subsequently struggled online, despite being composed of largely the same people. This can perhaps be explained by a sense of loss that accompanied the online transition [15], particularly for groups that adhered strongly to the ethos of the HVM and placed great emphasis on creating a distinct, user-led environment. Although not a replacement for nonverbal cues, future online groups may benefit from verbalizing the nonverbal: for example, by emphasizing the distinct values at the start of each group, acknowledging when a nongroup member appears in the background, or expressing that one wishes they could give another member a hug.

The distinct discursive elements of online groups were similarly consistent with previous research. For example, Weinberg [15] contended that there can be an unnaturally linear flow of dialogue in online psychotherapy groups because participants are in their own “boxes” and less able to pick up on nonverbal cues that indicate when the next person can speak. Similarly, facilitators in this study found that not only did discussions tend to focus on one person at a time rather than flow naturally between participants but disclosure was also not always as personal as in the face-to-face groups. This is a potentially important barrier to cohesion, and previous research suggests that facilitators can overcome this challenge by verbalizing statements and emotions, which previously would have remained nonverbal as a means of enhancing the continuity of dialogue between participants [35].

Although facilitators often noted significant challenges to group cohesion, many were nonetheless able to run successful and cohesive HVGs online. While previous research recommends acknowledging members’ subjective environment in moments where the space is disturbed (eg, a parent walks into the room during the group [15]), HVG facilitators went further and highlighted the utility of using each member’s individual space as a way of proactively showing interest and investment in their world. Given their open structure [30] and nonmanualized approach, HVGs may be particularly well-suited for this endeavor, as aspects of a member’s home environment can be incorporated more seamlessly and spontaneously into group discussion. Furthermore, previous literature recommends the use of “ice breaker” activities at the beginning of the group to help individuals ease into the space and connect with one another [36]. Sharing items from one’s home may serve as way of having less vulnerable conversations while still enabling members to connect with one another and gain unique insight into their subjective worlds. Similarly, HVG facilitators note that either planned (eg, sharing a video on voice hearing) or spontaneous activities (eg, members making music together) can be a useful tool in establishing cohesion. In this respect, facilitators in this study also emphasized the importance of capitalizing on moments of humor and spontaneity as a means of fostering shared experience between members. Although previous research has supported an association between humor and therapeutic alliance [37], this is the first study to specifically outline its utility in peer support groups and highlight how the unshared environment may lend itself more easily to these spontaneous moments. When moments of humor or spontaneity do arise, future groups should therefore purposefully use these moments to strengthen connections between members.

Mental health services may face particular challenges in providing adequate support to individuals who hear voices [17,18]. Therefore, despite the challenges outlined, caution should be taken in either interpreting online HVGs as inferior forms of support or in minimizing the value of their accessibility. Specifically, online groups allow members to engage more easily in ways that felt comfortable for them, as well as offering support to those who cannot access face-to-face groups. Furthermore, it was notable that facilitators did not mention voices and associated experiences (eg, paranoid beliefs, particularly those about being surveilled by technology) as being a major barrier to either group cohesion or to individuals successfully accessing the online space.

A significant strength of this study is its international scope, being the first of its kind to recruit participants throughout Europe and North America in a single group. Furthermore, none of the findings were region specific, meaning facilitators from across the global north may potentially benefit from implementing the strategies recommended below. Although HVGs are distinct in their ethos, the findings and recommendations may also be applicable to other forms of online support (although in non-HVG spaces, care should be taken to emphasize how the latter may be distinct from other types of mental health support groups). However, it should also be noted that the findings cannot be uncritically applied to groups facilitated in the global south, where norms and expectations about relational dynamics in peer support spaces may differ [38] and further research would be required.

Finally, the study must be interpreted in view of its limitations. Although previous research has highlighted the centrality of group relationships to the successful running of HVGs [6,7,9], the concept of group cohesion arises from psychotherapy literature rather than the peer support literature, and therefore cannot be uncritically applied [13]. Furthermore, the sample was racially homogeneous, with most participants identifying as White. Given the global spread of HVGs, future studies would be greatly enhanced by recruiting a more ethnically representative sample. Furthermore, 87% (7/8) of the facilitators with online experience had to adapt a face-to-face group to an online platform following the COVID-19 pandemic, and additional research should focus on delineating the development of cohesion in groups that started online compared to those that had to transition into being so. Finally, although efforts were made to member check results with all participants, responses were only received from 2 individuals (who suggested no substantial changes), and future research would be strengthened from incorporating a wider range of reflections into the analysis.

### Conclusions

In conclusion, it is possible to overcome the distinct challenges inherent to the medium to deliver cohesive HVGs online. Facilitators should be mindful that there are both nonverbal and discursive barriers to cohesion, many of which originate from the distance and detachment inherent in the online space. However, group cohesion may be enhanced by highlighting the distinct nature of the group, creating transitional space, verbalizing the nonverbal, capitalizing on spontaneity, and using participants’ unique environments to foster intimacy. Recommendations for promoting cohesive groups online can be found in Textbox 1, and it is hoped such forums may continue to prove an accessible and implementable form of support not only for individuals who hear voices but also for anyone who may benefit from peer engagement.

**Recommendations for optimizing group cohesion in online hearing voices peer support groups.**

**Recommendations**

- Avoid admitting all participants into the meeting at once. If possible, start the meeting 10-15 minutes before the designated start time and allow participants to arrive early to allow time to settle into the group space.
- Plan time for small-talk opportunities before the group starts and after it ends.
- Intentionally begin the group by reminding participants of its norms and values. This helps to differentiate the group from other online spaces.
- Use the environment by having participants share objects from their homes. Where participants have their cameras off, consider using auditory materials such as music.
- Be mindful of the flow of conversation; encourage participants to respond to one another rather than the facilitator.
- Use group activities, either psychoeducational (eg, sharing information or videos) or social (eg, everyone sharing an object from their environment) where appropriate.
- When interruptions occur (eg, a family member appears in the background), acknowledge and talk about it with the group.
- Frequently return to conversations about group norms and expectations (eg, is it acceptable to be multitasking while in the group?).
- Have refreshment breaks and encourage participants to bring food and drink back to the group the same way they would in face-to-face meetings.
- Capitalize on moments of humor and spontaneity by encouraging other members to join in and not moving to another topic too quickly.

## Appendix

Multimedia Appendix 1 COREQ (Consolidated Criteria for Reporting Qualitative Research) checklist.

Multimedia Appendix 2 Full participant characteristics.

## Abbreviations

COREQ: Consolidated Criteria for Reporting Qualitative Research
HVG: hearing voices peer support group
HVM: Hearing Voices Movement
PPIE: patient and public involvement and engagement
RTA: reflexive thematic analysis

## Data Availability

All data generated and analyzed during this study are not publicly available due to privacy and ethical restrictions.
