# Supplementary material for: Group Cohesion and Necessary Adaptations in Online Hearing Voices Peer Support Groups: Qualitative Study With Group Facilitators
Source: JMIR Form Res. 2024 May 3;8:e51694. doi: 10.2196/51694 (PMC11102034; doi:10.2196/51694)
Supplement: Multimedia Appendix 2 [file formative_v8i1e51694_app2.docx]

**Multimedia Appendix 2**. Full participant characteristics

| Name | Age (years) | Gender | Ethnicity | Geographical location | Group medium | Group moved online during the COVID-19 pandemic? | Length of time facilitating (years) | Frequency of group | Lived experience of voice hearing |
| --- | --- | --- | --- | --- | --- | --- | --- | --- | --- |
| Sean | 41 | Man | White | Western Europe | Face-to-face | No | 9 | Fortnightly | Yes |
| Annika | 34 | Woman | White | Northern Europe | Both | Yes | 4 | Weekly | Yes |
| Patrick | 46 | Man | White | Western Europe | Online | No | 1 | Weekly | Yes |
| Rachel | 25 | Woman | White | North America | Face-to-face | Yes | 1 | Weekly | Yes |
| Arjun | 36 | Man | South Asian | Western Europe | Both | Yes | 5 | Weekly | Yes |
| Callum | 52 | Woman | White | Western Europe | Both | Yes | 2 | Weekly | Yes |
| Sabina | 36 | Woman/gender fluid | White | Western Europe | Both | Yes | 8 | Fortnightly | Yes |
| Michail | 46 | Man | White | North America | Both | Yes | 5 | Weekly | No |
| Lex | 46 | Man | White | Western Europe | Both | Yes | 10 | Fortnightly | Yes |
| Noah | 47 | Man | White | Western Europe | Face-to-face | No | 8 | Fortnightly | Yes |
| Isabella | 45 | Woman | White | Western Europe | Both | Yes | 7 | Fortnightly | No |
